# Synthesis and crystal structure of *catena*-poly[[di­bromido­zinc(II)]-μ-2,3-di­methyl­pyrazine-κ^2^*N*^1^:*N*^4^]

**DOI:** 10.1107/S2056989025007613

**Published:** 2025-09-05

**Authors:** Christian Näther, Gaurav Bhosekar

**Affiliations:** a Institut für Anorganische Chemie, Universität Kiel, Max-Eyth.-Str. 2, 24118 Kiel, Germany; b Suman Ramesh Tulsiani Technical Campus - Faculty of Engineering, Pune, India; Institute of Chemistry, Chinese Academy of Sciences

**Keywords:** crystal structure, coordination polymer, synthesis, zinc bromide, 2,3-di­methyl­pyrazine

## Abstract

The title compound, [ZnBr_2_(C_6_H_8_N_2_)_2_]_*n*_, was prepared by the reaction of zinc bromide with 2,3-di­methyl­pyrazine in aceto­nitrile and is isotypic to the corresponding compound with zinc chloride reported recently [Näther & Bhosekar (2025 ▸). *Acta Cryst.* E**4**, 813–820]. The asymmetric unit consists of one zinc cation, two crystallographically independent bromide anions and one 2,3-di­methyl­pyrazine ligand, all of which are located in general positions. In the crystal, the Zn cations are tetra­hedrally coordinated by two bromide anions and two 2,3-di­methyl­pyrazine ligands and are linked by bridging 2,3-di­methyl­pyrazine ligands into corrugated chains that proceed along the *c*-axis direction. Measurements using powder X-ray diffraction show that a pure crystalline phase was obtained.

## Abstract 2

ZnBr_2_(2,3-di­methyl­pyrazine) is reported as isotypic to its zinc chloride counterpart. In the crystal, the Zn cations are connected by the 2,3-di­methyl­pyrazine ligands into corrugated chains.

## 1. Chemical context

We have been inter­ested in the synthesis and structures of transition-metal halide and pseudo halide compounds with N-donor coligands for a very long time because they show a versatile coordination and structural behavior. This is especially the case for compounds based on Cu^I^, in which the metal cations forms mono or dinuclear complexes or they are linked by the halide or pseudo halide anions into one-dimensional or two-dimensional networks (Näther *et al.*, 2001 ▸, 2002 ▸; Kromp & Sheldrick, 1999 ▸; Peng *et al.*, 2010 ▸; Li *et al.*, 2005 ▸). In most cases, for a given copper(I) halide or pseudo halide and a given coligand, compounds of different stoichiometry are observed, which include coligand-rich and coligand-deficient compounds. In this context we have found that the coligand-rich compounds can be transformed into the corresponding coligand-deficient compounds by heating (Näther & Jess, 2002 ▸, 2004 ▸).

In contrast to copper(I), compounds with twofold positively charged cations such as, for example, Zn^II^ or Cd^II^, show a limited number of *MX*_2_ networks (*M* = Zn, Cd). In these structures a tetra­hedral and also an octa­hedral coordination can be observed. The former coordination dominates for Zn^II^, whereas the latter is frequently found for Cd^II^ (Neumann *et al.*, 2018*a* ▸,*b* ▸). For Zn^II^ mainly discrete complexes are observed, whereas for Cd^II^ examples are known in which the metal cations are linked into chains (Neumann *et al.*, 2018*a* ▸,*b* ▸). The *MX*_2_ complexes or chains can additionally be connected if bridging coligands like pyrazine are used, and several such compounds are reported in the literature (Bailey & Pennington, 1997 ▸; Pickardt & Staub, 1997 ▸; Bhosekar *et al.*, 2006 ▸; Bourne *et al.*, 2001 ▸; Song *et al.*, 2004 ▸). Based on these observations, we prepared new ZnCl_*2*_ compounds with 2,3-di­methyl­pyrazine as ligand with the composition ZnCl_2_(2,3-di­methyl­pyrazine) and ZnCl_2_(2,3-di­methyl­pyrazine)_2_ (Näther & Bhosekar, 2025 ▸). In both compounds the Zn cations are tetra­hedrally coordinated, leading to the formation of discrete complexes in the 2,3-di­methyl­pyrazine-rich compound, whereas in the 2,3-di­methyl­pyrazine-deficient compounds the Zn cations are linked into chains. Very recently a corresponding compound with the composition ZnBr_2_(2,3-di­methyl­pyrazine)_2_ was reported that is isotypic to ZnCl_2_(2,3-di­methyl­pyrazine)_2_ and which was investigated for its photophysical properties (Yang *et al.*, 2025 ▸). Based on these results, we assumed that a further compound with ZnBr_2_ with the composition ZnBr_2_(2,3-di­methyl­pyrazine) could be prepared that might be isotypic to its ZnCl_2_ analog. Therefore, zinc bromide was reacted with equivalent amounts of 2,3-di­methyl­pyrazine and the crystals obtained were characterized by X-ray single crystal and powder diffraction.
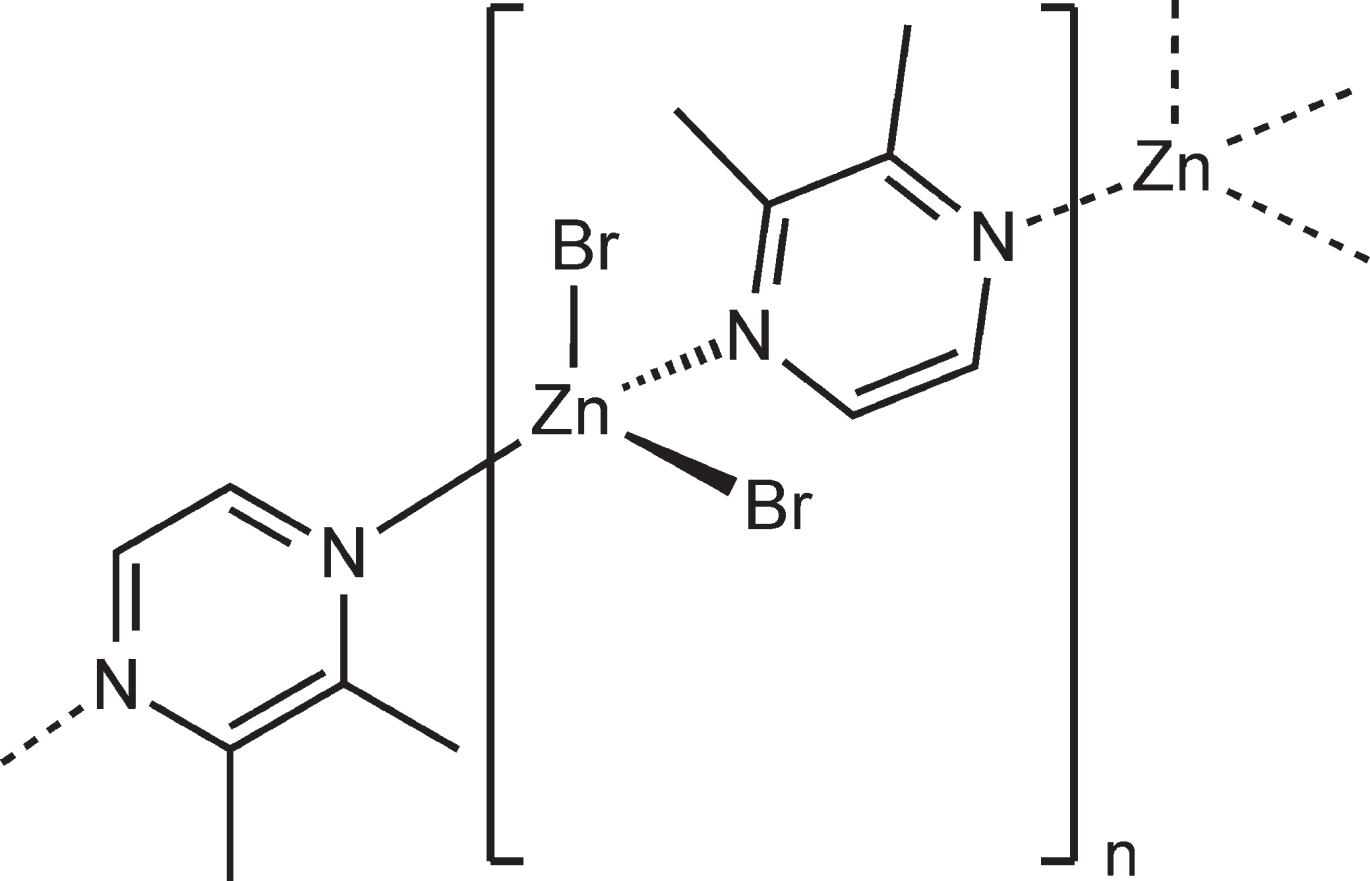


## 2. Structural commentary

**Figure 1:**
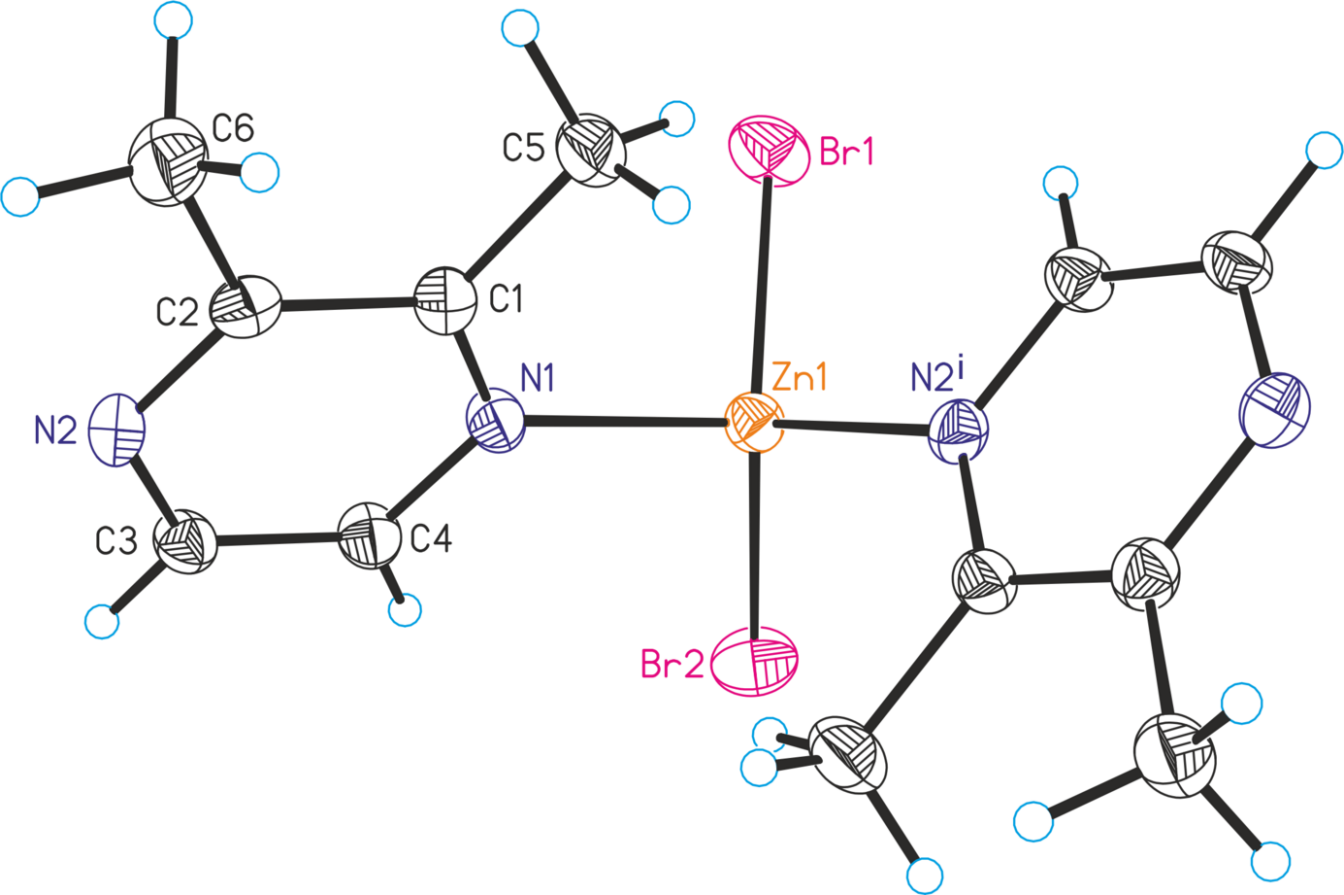
Crystal structure of the title compound with atom labeling and displacement ellipsoids drawn at the 50% probability level. Symmetry code for the generation of equivalent atoms: (i) −*x* + *y* + 1, −*x* + 1, *z* − 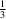.

**Figure 2:**
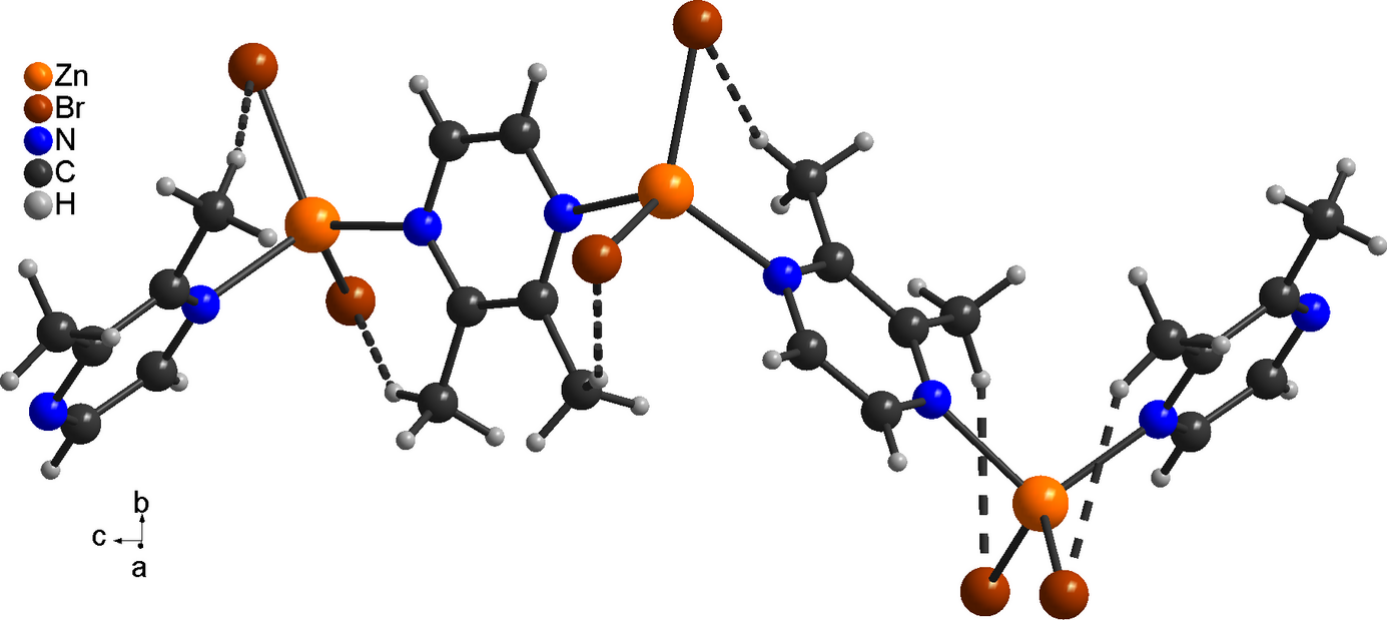
Fragment of the extended structure of the title compound with a view of part of a chain and intra­chain C⋯H—Br hydrogen bonding shown as dashed lines.

The new compound ZnBr_2_(2,3-di­methyl­pyrazine) is isotypic to the corresponding chloride compound ZnCl_2_(2,3-di­methyl­pyrazine) reported recently (Näther & Bhosekar, 2025 ▸). The asymmetric unit of the title compound consists of one Zn cation, two crystallographically independent bromide anions and one crystallographically independent 2,3-di­methyl­pyrazine ligand in general positions (Fig. 1 ▸). The Zn cations are fourfold coordinated by two N atoms of the 2,3-di­methyl­pyrazine coligands and two bromide anions in a distorted tetra­hedral geometry. The N—Zn—Br angles deviate only slightly from the ideal tetra­hedral values, whereas the Br—Zn—Br angles are larger and the N—Zn—N angles are smaller (Table 1 ▸), presumably because of steric repulsion between the halide anions. The metal cations are linked into chains along the crystallographic *c*-axis direction by bridging 2,3-di­methyl­pyrazine ligands (Fig. 2 ▸). These chains are corrugated because of the tetra­hedral coordination (Fig. 2 ▸).

## 3. Supra­molecular features

**Figure 3:**
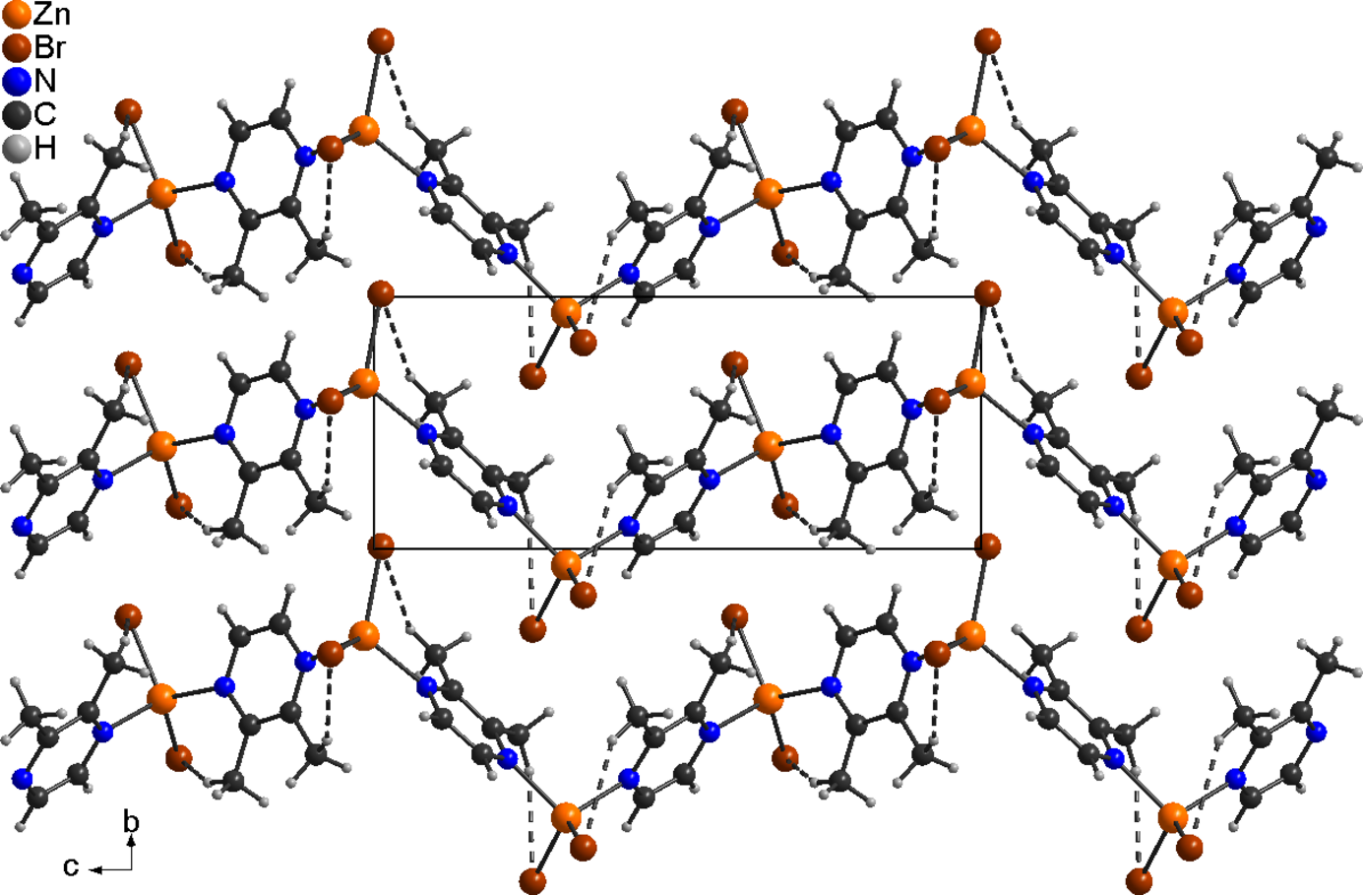
Crystal structure of the title compound with a view along the crystallographic *a*-axis direction and intra­chain C⋯H—Br hydrogen bonding shown as dashed lines.

In the crystal, intra­chain C—H⋯Br hydrogen bonding with C—H⋯Br angles of 151 and 150° is observed (Fig. 2 ▸ and Table 2 ▸). These chains are linked by inter­chain C—H⋯Br contacts that show much smaller angles and thus should represent only very weak inter­actions (Fig. 3 ▸ and Table 2 ▸).

## 4. Database survey

As mentioned above, the title compound is isotypic to the corresponding 2,3-di­methyl­pyrazine compound with ZnCl_2_ already reported in the literature (Näther & Bhosekar, 2025 ▸). There are two additional compounds with the composition ZnCl_2_(2,3-di­methyl­pyrazine) (Näther & Bhosekar, 2025 ▸) and ZnBr_2_(2,3-di­methyl­pyrazine) (Yang *et al.*, 2025 ▸) that are isotypic and that are built up of discrete complexes with a tetra­hedral Zn coordination. Further compounds with twofold positively charged transition-metal halides and 2,3-di­methyl­pyrazine as ligand are not reported in the CCDC database (CSD Version 5.43, January 2025; Groom *et al.*, 2016 ▸) using CONQUEST (Bruno *et al.*, 2002 ▸). However, some compounds are known with the unsubstituted ligand pyrazine. These include Cd*X*_2_(pyrazine) [*X* = Cl, Refcode TISSUJ ( (Pickardt & Staub, 1997 ▸)] ), *X* = Br, RINSIQ and RINSOW (Bailey & Pennington, 1997 ▸); *X* = I, RINSIQ01 and RINSOW01 (Pickardt & Staub, 1997 ▸)] in which the metal cations are octa­hedrally coordinated and are linked by pairs of halide anions into chains that are further connected into layers by the pyrazine ligands.

More compounds are reported with Zn^II^ cations and pyrazine. In contrast to the title compound with 2,3-di­methyl­pyrazine as coligand, these compounds show an octa­hedral coordination. This is the case in, *e.g.*, ZnCl_2_(pyrazine)_2_ (Refcode REMPAB; Bhosekar *et al.*, 2006 ▸) in which the Zn cations are linked into layers by the pyrazine ligands. In the pyrazine-deficient compound ZnCl_2_(pyrazine) (Refcode TISTAQ; Pickardt & Staub, 1997 ▸), the Zn cations are linked into chains by pairs of bridging chloride anions that are further connected into layers by the pyrazine ligands. With ZnBr_2_, two compounds are known of which the pyrazine-rich compound ZnBr_2_(pyrazine)_2_ crystallizes in two modifications with layered networks of the same topology. One of them is isotypic to the corresponding chloride compound [Refcodes EBOLAI (Bourne *et al.*, 2001 ▸) and EBOLAI01 (Bhosekar *et al.*, 2006 ▸)]. They also include ZnBr_2_(pyrazine), which crystallizes differently from the chloride compound (Refcode EBOKUB; Bourne *et al.*, 2001 ▸). Finally, ZnI_2_(pyrazine) is reported that shows a structure similar to that of the bromide compound [Refcodes ISOPOV (Song *et al.*, 2004 ▸) and ISOPOV01 (Bhosekar *et al.*, 2006 ▸)].

## 5. Synthesis and crystallization

Zinc bromide and 2,3-di­methyl­pyrazine were purchased from Sigma-Aldrich.

**Synthesis**

0.5 mmol (112.6 mg) of zinc bromide were reacted with 0.5 mmol (54.1 mg) of 2,3-di­methyl­pyrazine in 1 mL of aceto­nitrile. The reaction mixture was stirred for 3 d and the precipitate was filtered off and dried. Single crystals were obtained using the same ratio of reactants without stirring.

**Figure 4:**
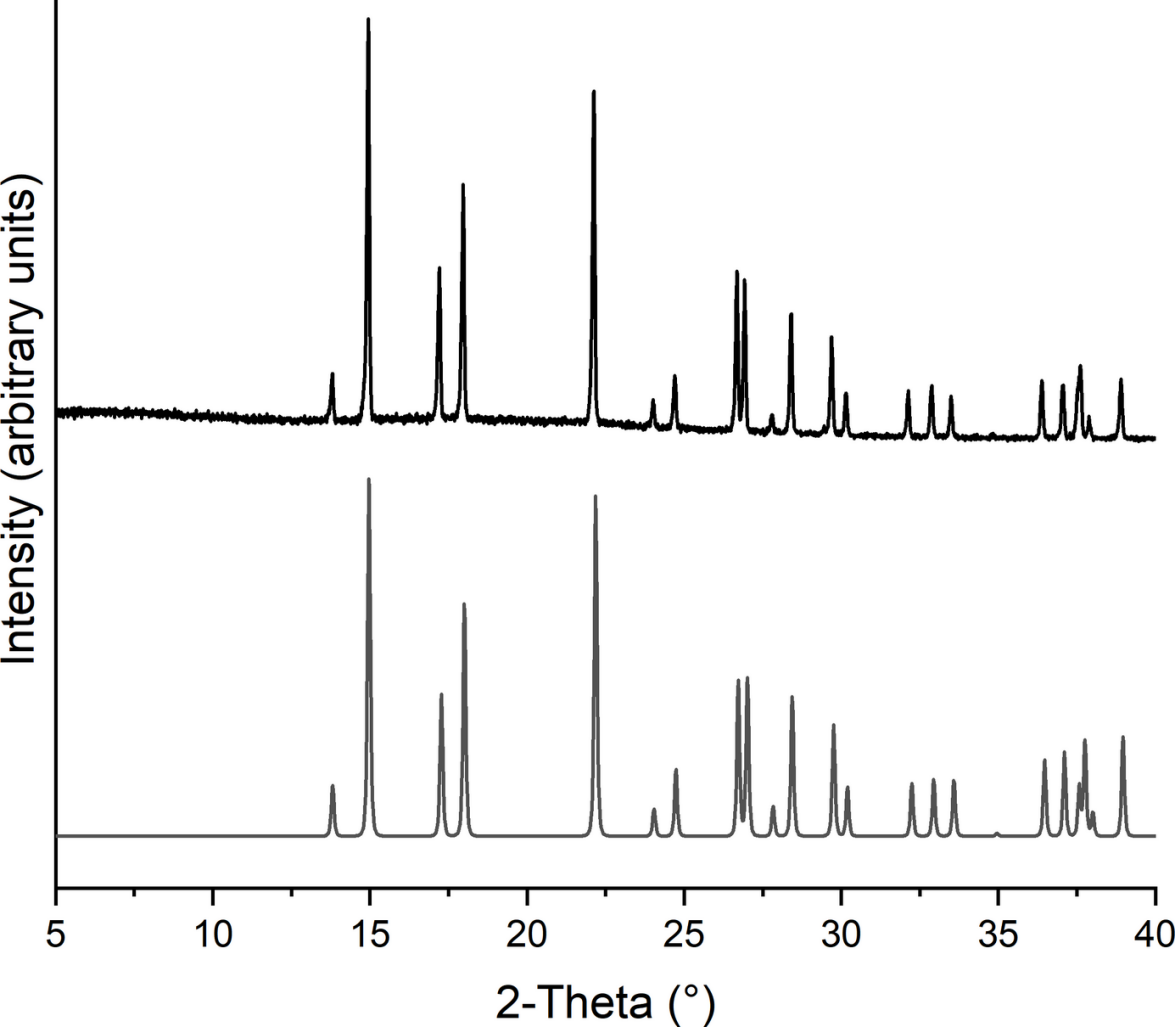
Experimental (top) and calculated (bottom) X-ray powder pattern of the title compound.

The title compound was additionally investigated by X-ray powder diffraction, which shows that a pure sample has been obtained (Fig. 4 ▸).

**Experimental details**

The PXRD measurements were performed with Cu *Kα*_1_ radiation (λ = 1.540598 Å) using a Stoe Transmission Powder Diffraction System (STADI P) that is equipped with a MYTHEN 1K detector and a Johansson-type Ge(111) monochromator.

## 6. Refinement

Crystal data, data collection and structure refinement details are summarized in Table 3 ▸. Hydrogen atoms were positioned with idealized geometry (methyl H atoms allowed to rotate but not to tip) and were refined isotropically with *U*_iso_(H) = 1.2 *U*_eq_(C) (1.5 for methyl H atoms).

## Supplementary Material

Crystal structure: contains datablock(s) I, global. DOI: 10.1107/S2056989025007613/nx2028sup1.cif

Structure factors: contains datablock(s) I. DOI: 10.1107/S2056989025007613/nx2028Isup2.hkl

CCDC reference: 2482737

Additional supporting information:  crystallographic information; 3D view; checkCIF report

## Figures and Tables

**Table 1:** Selected geometric parameters (Å, °)

Zn1—N2^i^	2.085 (6)	Zn1—Br1	2.3380 (12)
Zn1—N1	2.118 (7)	Zn1—Br2	2.3501 (12)
			
N2^i^—Zn1—N1	103.1 (3)	N2^i^—Zn1—Br2	115.18 (19)
N2^i^—Zn1—Br1	109.2 (2)	N1—Zn1—Br2	108.13 (19)
N1—Zn1—Br1	105.25 (19)	Br1—Zn1—Br2	114.87 (5)

Symmetry code: (i) 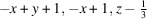.

**Table 2:** Hydrogen-bond geometry (Å, °)

*D*—H⋯*A*	*D*—H	H⋯*A*	*D*⋯*A*	*D*—H⋯*A*
C3—H3⋯Br1^ii^	0.94	2.95	3.604 (8)	128
C3—H3⋯Br2^iii^	0.94	2.99	3.687 (8)	132
C4—H4⋯Br2	0.94	2.95	3.605 (8)	128
C5—H5*C*⋯Br1	0.97	2.95	3.824 (10)	151
C6—H6*A*⋯Br1^iv^	0.97	2.99	3.710 (10)	132
C6—H6*C*⋯Br2^ii^	0.97	2.95	3.820 (10)	150

Symmetry codes: (ii) 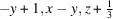; (iii) 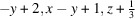; (iv) 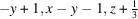.

**Table 3:** Experimental details

Crystal data	
Chemical formula	[ZnBr_2_(C_6_H_8_N_2_)_2_]
*M*_r_	333.33
Crystal system, space group	Trigonal, *P*3_1_
Temperature (K)	220
*a*, *c* (Å)	7.3972 (3), 15.3874 (8)
*V* (Å^3^)	729.17 (7)
*Z*	3
Radiation type	Mo *K*α
μ (mm^−1^)	10.69
Crystal size (mm)	0.11 × 0.07 × 0.06

Data collection	
Diffractometer	Stoe *IPDS1*
Absorption correction	Numerical (*X-RED* and *X-SHAPE*; Stoe, 2008 ▸)
*T*_min_, *T*_max_	0.067, 0.156
No. of measured, independent and observed [*I* > 2σ(*I*)] reflections	7080, 2343, 2231
*R*_int_	0.076
(sin θ/λ)_max_ (Å^−1^)	0.660

Refinement	
*R*[*F*^2^ > 2σ(*F*^2^)], *wR*(*F*^2^), *S*	0.040, 0.102, 1.06
No. of reflections	2343
No. of parameters	103
No. of restraints	1
H-atom treatment	H-atom parameters constrained
Δρ_max_, Δρ_min_ (e Å^−3^)	0.71, −0.73
Absolute structure	Flack *x* determined using 1049 quotients [(*I*^+^)−(*I*^−^)]/[(*I*^+^)+(*I*^−^)] (Parsons *et al.*, 2013 ▸)
Absolute structure parameter	0.01 (2)

Computer programs: *X-AREA* (Stoe, 2008 ▸), *SHELXT* (Sheldrick, 2015*a* ▸), *SHELXL* (Sheldrick, 2015*b* ▸), *DIAMOND* (Brandenburg, 1999 ▸) and *XP* in *SHELXTL-PC* (Sheldrick, 2008 ▸) and *publCIF* (Westrip, 2010 ▸).

## supplementary crystallographic information

### catena-Poly[[dibromidozinc(II)]-µ-2,3-dimethylpyrazine-κ2N1:N4] Crystal data

**Table:**

[ZnBr_2_(C_6_H_8_N_2_)_2_]	*D*_x_ = 2.277 Mg m^−^^3^
*M**_r_* = 333.33	Mo *K*α radiation, λ = 0.71073 Å
Trigonal, *P*3_1_	Cell parameters from 7962 reflections
*a* = 7.3972 (3) Å	θ = 10.4–27.1°
*c* = 15.3874 (8) Å	µ = 10.69 mm^−^^1^
*V* = 729.17 (7) Å^3^	*T* = 220 K
*Z* = 3	Block, light yellow
*F*(000) = 474	0.11 × 0.07 × 0.06 mm

### catena-Poly[[dibromidozinc(II)]-µ-2,3-dimethylpyrazine-κ2N1:N4] Data collection

**Table:**

Stoe IPDS-1 diffractometer	2231 reflections with *I* > 2σ(*I*)
Phi scans	*R*_int_ = 0.076
Absorption correction: numerical (X-Red and X-Shape; Stoe, 2008)	θ_max_ = 28.0°, θ_min_ = 3.2°
*T*_min_ = 0.067, *T*_max_ = 0.156	*h* = −9→9
7080 measured reflections	*k* = −9→9
2343 independent reflections	*l* = −20→20

### catena-Poly[[dibromidozinc(II)]-µ-2,3-dimethylpyrazine-κ2N1:N4] Refinement

**Table:**

Refinement on *F*^2^	H-atom parameters constrained
Least-squares matrix: full	*w* = 1/[σ^2^(*F*_o_^2^) + (0.0647*P*)^2^ + 0.5878*P*] where *P* = (*F*_o_^2^ + 2*F*_c_^2^)/3
*R*[*F*^2^ > 2σ(*F*^2^)] = 0.040	(Δ/σ)_max_ < 0.001
*wR*(*F*^2^) = 0.102	Δρ_max_ = 0.71 e Å^−^^3^
*S* = 1.06	Δρ_min_ = −0.73 e Å^−^^3^
2343 reflections	Extinction correction: SHELXL-2016/6 (Sheldrick 2015b), Fc^*^=kFc[1+0.001xFc^2^λ^3^/sin(2θ)]^-1/4^
103 parameters	Extinction coefficient: 0.043 (4)
1 restraint	Absolute structure: Flack *x* determined using 1049 quotients [(*I*^+^)-(*I*^-^)]/[(*I*^+^)+(*I*^-^)] (Parsons *et al.*, 2013)
Hydrogen site location: inferred from neighbouring sites	Absolute structure parameter: 0.01 (2)

### catena-Poly[[dibromidozinc(II)]-µ-2,3-dimethylpyrazine-κ2N1:N4] Special details

**Table:**

Geometry. All esds (except the esd in the dihedral angle between two l.s. planes) are estimated using the full covariance matrix. The cell esds are taken into account individually in the estimation of esds in distances, angles and torsion angles; correlations between esds in cell parameters are only used when they are defined by crystal symmetry. An approximate (isotropic) treatment of cell esds is used for estimating esds involving l.s. planes.

### catena-Poly[[dibromidozinc(II)]-µ-2,3-dimethylpyrazine-κ2N1:N4] Fractional atomic coordinates and isotropic or equivalent isotropic displacement parameters (Å^2^)

**Table:**

	*x*	*y*	*z*	*U*_iso_*/*U*_eq_	
Zn1	1.06358 (14)	0.65633 (14)	0.01689 (5)	0.0206 (3)	
Br1	1.31606 (14)	0.58557 (16)	0.07229 (6)	0.0339 (3)	
Br2	1.18263 (16)	1.01071 (14)	−0.01098 (6)	0.0364 (3)	
N1	0.8300 (11)	0.5540 (10)	0.1140 (4)	0.0214 (13)	
C1	0.7097 (13)	0.3550 (13)	0.1380 (5)	0.0218 (14)	
C2	0.5591 (13)	0.3036 (12)	0.2051 (5)	0.0209 (14)	
N2	0.5454 (10)	0.4553 (11)	0.2470 (4)	0.0203 (12)	
C3	0.6710 (12)	0.6539 (12)	0.2219 (5)	0.0217 (14)	
H3	0.662006	0.761430	0.250495	0.026*	
C4	0.8122 (13)	0.7038 (12)	0.1554 (5)	0.0222 (15)	
H4	0.896891	0.844010	0.138842	0.027*	
C5	0.7352 (15)	0.1869 (14)	0.0961 (6)	0.0286 (17)	
H5A	0.644589	0.133729	0.045754	0.043*	
H5B	0.698387	0.074531	0.137308	0.043*	
H5C	0.879174	0.243448	0.078088	0.043*	
C6	0.4191 (16)	0.0817 (14)	0.2324 (7)	0.0322 (19)	
H6A	0.502059	0.028064	0.258741	0.048*	
H6B	0.346705	−0.002001	0.181996	0.048*	
H6C	0.317974	0.075293	0.274258	0.048*	

### catena-Poly[[dibromidozinc(II)]-µ-2,3-dimethylpyrazine-κ2N1:N4] Atomic displacement parameters (Å^2^)

**Table:**

	*U*^11^	*U*^22^	*U*^33^	*U*^12^	*U*^13^	*U*^23^
Zn1	0.0210 (4)	0.0201 (4)	0.0207 (4)	0.0102 (3)	−0.0004 (3)	−0.0012 (3)
Br1	0.0299 (4)	0.0399 (5)	0.0389 (5)	0.0228 (4)	−0.0114 (4)	−0.0094 (4)
Br2	0.0393 (5)	0.0218 (4)	0.0445 (6)	0.0126 (4)	0.0007 (4)	0.0070 (4)
N1	0.022 (3)	0.025 (3)	0.020 (3)	0.014 (3)	0.001 (2)	−0.001 (3)
C1	0.023 (4)	0.023 (3)	0.020 (3)	0.012 (3)	0.002 (3)	0.000 (3)
C2	0.023 (3)	0.018 (3)	0.022 (3)	0.010 (3)	−0.002 (3)	0.002 (3)
N2	0.020 (3)	0.022 (3)	0.017 (3)	0.009 (3)	0.003 (2)	0.000 (2)
C3	0.022 (3)	0.018 (3)	0.025 (3)	0.010 (3)	0.003 (3)	0.001 (3)
C4	0.024 (3)	0.016 (3)	0.025 (4)	0.008 (3)	0.003 (3)	0.000 (3)
C5	0.037 (4)	0.024 (4)	0.028 (4)	0.018 (3)	0.008 (4)	0.002 (3)
C6	0.037 (5)	0.022 (4)	0.033 (4)	0.011 (3)	0.011 (4)	0.005 (3)

### catena-Poly[[dibromidozinc(II)]-µ-2,3-dimethylpyrazine-κ2N1:N4] Geometric parameters (Å, º)

**Table:**

Zn1—N2^i^	2.085 (6)	N2—C3	1.344 (10)
Zn1—N1	2.118 (7)	C3—C4	1.374 (11)
Zn1—Br1	2.3380 (12)	C3—H3	0.9400
Zn1—Br2	2.3501 (12)	C4—H4	0.9400
N1—C1	1.336 (10)	C5—H5A	0.9700
N1—C4	1.340 (10)	C5—H5B	0.9700
C1—C2	1.424 (12)	C5—H5C	0.9700
C1—C5	1.494 (11)	C6—H6A	0.9700
C2—N2	1.341 (11)	C6—H6B	0.9700
C2—C6	1.498 (11)	C6—H6C	0.9700
			
N2^i^—Zn1—N1	103.1 (3)	N2—C3—C4	121.6 (7)
N2^i^—Zn1—Br1	109.2 (2)	N2—C3—H3	119.2
N1—Zn1—Br1	105.25 (19)	C4—C3—H3	119.2
N2^i^—Zn1—Br2	115.18 (19)	N1—C4—C3	120.5 (7)
N1—Zn1—Br2	108.13 (19)	N1—C4—H4	119.8
Br1—Zn1—Br2	114.87 (5)	C3—C4—H4	119.8
C1—N1—C4	119.5 (7)	C1—C5—H5A	109.5
C1—N1—Zn1	124.3 (5)	C1—C5—H5B	109.5
C4—N1—Zn1	116.1 (5)	H5A—C5—H5B	109.5
N1—C1—C2	119.7 (7)	C1—C5—H5C	109.5
N1—C1—C5	120.4 (7)	H5A—C5—H5C	109.5
C2—C1—C5	119.9 (7)	H5B—C5—H5C	109.5
N2—C2—C1	120.1 (7)	C2—C6—H6A	109.5
N2—C2—C6	118.8 (7)	C2—C6—H6B	109.5
C1—C2—C6	121.1 (7)	H6A—C6—H6B	109.5
C2—N2—C3	118.5 (7)	C2—C6—H6C	109.5
C2—N2—Zn1^ii^	124.8 (5)	H6A—C6—H6C	109.5
C3—N2—Zn1^ii^	116.7 (5)	H6B—C6—H6C	109.5
			
C4—N1—C1—C2	2.2 (11)	C6—C2—N2—C3	−179.8 (8)
Zn1—N1—C1—C2	179.1 (6)	C1—C2—N2—Zn1^ii^	−177.0 (6)
C4—N1—C1—C5	−176.6 (8)	C6—C2—N2—Zn1^ii^	0.8 (11)
Zn1—N1—C1—C5	0.3 (11)	C2—N2—C3—C4	−0.5 (12)
N1—C1—C2—N2	−3.3 (12)	Zn1^ii^—N2—C3—C4	179.0 (6)
C5—C1—C2—N2	175.4 (8)	C1—N1—C4—C3	−0.2 (12)
N1—C1—C2—C6	178.9 (8)	Zn1—N1—C4—C3	−177.4 (6)
C5—C1—C2—C6	−2.3 (12)	N2—C3—C4—N1	−0.7 (13)
C1—C2—N2—C3	2.4 (12)		

Symmetry codes: (i) −*x*+*y*+1, −*x*+1, *z*−1/3; (ii) −*y*+1, *x*−*y*, *z*+1/3.

### catena-Poly[[dibromidozinc(II)]-µ-2,3-dimethylpyrazine-κ2N1:N4] Hydrogen-bond geometry (Å, º)

**Table:**

*D*—H···*A*	*D*—H	H···*A*	*D*···*A*	*D*—H···*A*
C3—H3···Br1^ii^	0.94	2.95	3.604 (8)	128
C3—H3···Br2^iii^	0.94	2.99	3.687 (8)	132
C4—H4···Br2	0.94	2.95	3.605 (8)	128
C5—H5*C*···Br1	0.97	2.95	3.824 (10)	151
C6—H6*A*···Br1^iv^	0.97	2.99	3.710 (10)	132
C6—H6*C*···Br2^ii^	0.97	2.95	3.820 (10)	150

Symmetry codes: (ii) −*y*+1, *x*−*y*, *z*+1/3; (iii) −*y*+2, *x*−*y*+1, *z*+1/3; (iv) −*y*+1, *x*−*y*−1, *z*+1/3.
